# Health Economic Evaluation of Cognitive‐Behavioral Therapy for Adolescents With Binge‐Eating Disorder in Germany

**DOI:** 10.1002/eat.24413

**Published:** 2025-03-10

**Authors:** Nicolas Pardey, Ricarda Schmidt, Jan Zeidler, Anja Hilbert

**Affiliations:** ^1^ Center for Health Economics Research Hannover (CHERH) Leibniz University Hannover Hannover Germany; ^2^ Department of Psychosomatic Medicine and Psychotherapy, Behavioral Medicine Research Unit, Integrated Research and Treatment Center Adiposity Diseases University of Leipzig Medical Center Leipzig Germany

**Keywords:** adolescents, binge‐eating disorder, cognitive behavioral therapy, cost‐effectiveness, cost‐utility, incremental cost‐effectiveness ratio, quality‐adjusted life year

## Abstract

**Objective:**

To determine the cost‐effectiveness of cognitive‐behavioral therapy (CBT) for adolescents with binge‐eating disorder (BED), focusing on the costs per binge‐free episode and per quality‐adjusted life year (QALY) gained in comparison to a waitlist (WL) control group.

**Method:**

In the prospective, randomized superiority Binge‐Eating Disorder in Adolescents (BEDA) trial, evaluating the efficacy of CBT with 20 individual sessions over 4 months versus WL, clinical and cost data were assessed at baseline and after 4 months. Missing values were imputed using multiple imputation techniques. The incremental cost‐effectiveness ratio (ICER) was calculated. To reflect uncertainty, nonparametric bootstrapping was performed, and the results were presented in the form of cost‐effectiveness acceptability curves (CEACs).

**Results:**

The study population consisted of 73 adolescents (82.2% female, mean age: 15.5 ± 2.6 years). Participants receiving CBT (*n* = 37) exhibited 4.7 more binge‐free episodes (*p* = 0.0056) than the WL group (*n* = 36). The ICER was €46.70 for the gain of a binge‐free episode and €128,861 for the gain of a QALY.

**Discussion:**

The probability of cost‐effectiveness for achieving a binge‐free episode is > 95% at a willingness‐to‐pay of €101. In terms of QALYs, CBT for BED may be a cost‐effective intervention. A longer follow‐up period may have yielded more favorable cost‐effectiveness results.

**Trial Registration:**

German Clinical Trials Register, https://www.drks.de, DRKS00000542

**Summary**

- Cognitive‐behavioral therapy (CBT) for binge‐eating disorder (BED) in adolescents significantly increases binge‐free episodes compared with a waitlist (WL) control group over a four‐month intervention period.
- Additionally, CBT may improve quality of life, though these gains are not statistically significant.
- The intervention incurs moderate additional costs, suggesting CBT for BED may be a cost‐effective intervention.

## 1 Introduction

Binge‐eating disorder (BED) is a mental disorder characterized by recurrent, objectively large binge‐eating episodes without regular compensatory behaviors to prevent weight gain (American Psychiatric Association 2022; World Health Organization 2022). For adolescents, a developmentally sensitive, that is, age‐adapted, diagnosis of BED in children and adolescents has been proposed to include objective and subjective binge‐eating episodes (Bravender et al. 2010), characterized by a sense of loss of control (LOC) over eating an objectively or subjectively large amount of food. BED typically develops during adolescence and has a lifetime prevalence of 1.3% (Kjeldbjerg and Clausen 2023). Adolescents with BED exhibit impaired health‐related quality of life (HRQoL), as demonstrated by comparisons with the general population and adolescents with obesity without BED (Pasold et al. 2014; Ranzenhofer et al. 2012). Cognitive‐behavioral therapy (CBT) is currently the most well‐established approach for the treatment of BED in adults (Hilbert et al. 2019). Preliminary evidence indicates that CBT may also be an efficacious treatment for adolescents with BED (Hilbert et al. 2020; Debar et al. 2013; Mazzeo et al. 2016). Due to limited resources, services in socially financed healthcare systems should be not only efficacious but also cost‐effective.

Regarding its cost‐effectiveness, König et al. (2018) evaluated individual CBT versus therapist‐guided Internet‐based self‐help (GSH‐I) in adults with BED. The study reported an incremental cost‐effectiveness ratio (ICER) of €63 per additional binge‐free day gained by the CBT condition. However, the cost‐effectiveness of CBT in adolescents with BED remains unclear, as their lower use of the healthcare system results in reduced direct costs compared to adults. This analysis addresses this gap by presenting a health economic evaluation of a randomized controlled trial conducted by (Hilbert et al. 2020), evaluating individual CBT compared to a waitlist (WL) condition in adolescents with BED aged 12–20 years.

The ICER evaluates the costs and effects between an intervention and a control group. It specifically indicates the additional costs associated with CBT to achieve an additional unit of outcome compared to WL. In this study, the outcomes considered were either a binge‐free episode or a quality‐adjusted life year (QALY). The QALY concept integrates life expectancy and HRQoL. HRQoL is scored between 0 (death) and 1 (perfect health), with one QALY representing 1 year of life in perfect health. A QALY can reflect various combinations of life expectancy and HRQoL (e.g., two life years with a HRQoL of 0.5 each) (Glick et al. 2015).

There are four ICER scenarios: If CBT is more effective (generates more binge‐free episodes) and less costly, it dominates WL and should be preferred. If CBT is more effective but also more costly (or less effective and less costly), the cost‐effectiveness depends on the society's maximum willingness to pay per additional binge‐free episode. The willingness‐to‐pay is the maximum amount a payer, such as society or a social health insurance fund, is willing to spend to achieve an additional health outcome, like a binge‐free day. If CBT is less effective and more costly, CBT is dominated and should not be considered from an economic perspective (Drummond et al. 2015).

This health economic evaluation aimed to determine the cost‐effectiveness (cost per binge‐free episode) and the cost‐utility (cost per QALY gained) for CBT compared to the WL condition in adolescents with BED. The results may enable decision‐makers in healthcare to make an evidence‐based decision on the implementation of CBT for adolescents with BED in routine care (Weinstein et al. 2009).

## 2 Method

### 2.1 Study Design and Participants

The Binge‐Eating Disorder in Adolescents (BEDA) study is a single‐center, prospective, randomized superiority trial (German clinical trials register, no. DRKS00000542) that was conducted in 20 individual face‐to‐face, 50‐min sessions over 4 months. The intervention was conducted at Leipzig University Medical Center between 2013 and 2015 (for further methodological detail, see (Hilbert et al. 2020; Hilbert 2013)). Ethical approval for this study was obtained from the Ethical Committee at the University of Leipzig Medical Center.

All participants completed the assessments at baseline. The second measurement was conducted 4 months after randomization (post‐assessment). For the CBT group, this post‐assessment measurement represented the post‐treatment measurement. Subsequently, all WL patients were offered individual CBT. During the WL period, participants were instructed to refrain from seeking any additional medical or psychological treatment for BED or obesity. As the WL participated in the intervention after post‐assessment, the analysis focused on the post‐assessment measurement only, although a two‐year follow‐up after CBT was conducted (Hilbert et al. 2020).

### 2.2 Assessments

The cost‐effectiveness analysis focused on the number of binge‐free episodes over the past 28 days before baseline and post‐assessment. These were calculated as the difference in binge‐eating episodes between the CBT and WL groups. The number of binge‐eating episodes included objective and subjective binge‐eating episodes, according to the common definition of binge eating in youth (Tanofsky‐Kraff et al. 2020). The cost‐utility analysis assessed the QALYs gained for each group during the four‐month intervention/waiting period. In this study, HRQoL was assessed through the Short Form 12 (SF‐12) questionnaire (Ware et al. 1995) covering physical and mental health. The assessment of QALYs was conducted via the Short Form 6 Dimensions (SF‐6D), which is a preference‐based instrument calculated from the SF‐12 (University of Sheffield n.d.). The differences in costs and effects were calculated according to the time horizon of the cost‐effectiveness and cost‐utility analyses.

### 2.3 Costs

The cost variable encompasses both the expenditure incurred by participants for the utilization of healthcare services and the intervention costs. Health resources utilization was assessed using an age‐adapted version of the Client Sociodemographic and Service Receipt Inventory (Roick et al. 2001). The questionnaire requests information regarding the type and frequency of healthcare utilization over a period of 6 months prior to the baseline assessment and 2 months prior to the post‐assessment. Utilization was measured in terms of the frequency of contacts with general practitioners and specialists, the length of hospital stays and rehabilitation facilities, non‐physician services (e.g., physiotherapy), and the medication taken. The costs of all healthcare services were calculated using standard costs applicable to the German healthcare system (Bock et al. 2014). The term “standard costs” refers to the average costs incurred for the utilization of a healthcare service, such as a visit to a general practitioner. Medication costs were calculated using the German prescription report, with net costs per defined daily dose (DDD) (Ludwig et al. 2022). This study presents data on direct medical costs exclusively. Intervention costs represent the billing rate of a psychological psychotherapist, which is €19.09 per 10 min in 2025 costs, resulting in a rate of €95.45 for a 50‐min session (Kassenärztliche Bundesvereinigung 2025). CEACs for a billing rate considering 2015 costs are shown in the Supporting Information.

### 2.4 Statistical Analysis

Missing post‐assessment values for SF‐6D scores, binge‐eating episodes, and costs from healthcare utilization were imputed using predictive mean matching (PMM). In PMM, values are imputed using data from individuals with complete data and equivalent characteristics, including the treatment group, age, gender, and binge‐eating episodes or HRQoL at baseline (Faria et al. 2014). In the SF‐12 questionnaire, a value was considered missing if at least one item was unanswered. Due to the presence of missing data, we conducted PMM on binge‐eating episodes in 10 individuals (CBT: 24.3%, WL: 2.8%), as well as SF‐6D score imputation in 19 individuals (CBT: 35.1%, WL: 16.7%).

All *p*‐values were derived from the Mann–Whitney U test. The effect size was quantified using Pearson's *r*, with *r* = 0.1 indicating small, *r* = 0.3 medium, and *r* = 0.5 large effects. Two‐tailed *p*‐values < 0.05 were taken to indicate statistical significance. Data preparation was conducted with MS Excel 2016, and data analysis was performed using SAS, Version 9.4.

### 2.5 Sensitivity Analysis

As the ICER is a point estimate, non‐parametric bootstrapping was used to assess the robustness of the results (Briggs 2000). This was achieved by repeatedly drawing 10,000 random samples from the study population with replacement (Campbell and Torgerson 1999). The probability of cost‐effectiveness is dependent upon the distribution of the 10,000 samples. As a reliable method for accounting for the uncertainty of costs and effects in cost‐effectiveness analyses, non‐parametric bootstrapping is a suitable approach for this purpose (Polsky et al. 1997). Consequently, cost‐effectiveness acceptability curves (CEACs) were constructed, indicating the probability of cost‐effectiveness for a maximum willingness to pay for the gain of a binge‐free episode or a QALY.

## 3 Results

The sample comprised 73 adolescents (82.2% female, mean age of 15.5 ± 2.6 years, Table 1). No differences were obvious in the baseline characteristics between the CBT (*n* = 37) and WL (*n* = 36) groups. At post‐assessment, the CBT group exhibited a significantly higher number of binge‐free episodes (4.7 more) than the WL group (z = 2.77, *p* = 0.0056, *r* = 0.32). HRQoL was not significantly higher in the CBT versus WL group at post‐assessment (0.7681 vs. 0.7317, z = −1.31, *p* = 0.1897, r = 0.15). Total costs were observed to be higher in the CBT group (€1715.56 ± €626.30 vs. €837.48 ± €1270.68, *z* = −4.76, *p* = < 0.0001, *r* = 0.56), which was attributable to intervention costs (€1.320.82 ± €624.09). The ICER was €46.70 for gaining a binge‐free episode. In the cost‐utility analysis, the ICER for the gain of a QALY was €128,861.

**TABLE 1:** Participant's baseline characteristics and post‐assessment results.

	Baseline				Post‐assessment				Effect size (95%‐CI)^a^	*p*
	CBT		WL		CBT		WL			
	Mean or *n*	SD or %	Mean or *n*	SD or %	Mean or *n*	SD or %	Mean or *n*	SD or %		
Male	7	18.9	6	16.7						
Female	30	81.1	30	83.3						
Age (years)	15.5	2.5	15.6	2.7						
BMI (kg/m^2^)	28.2	4.9	29.1	6.7						
Binge‐Eating Episodes^b^	11.8	9.8	11.3	8.9	1.3	2.5	6.0	8.5	0.32 (0.10;0.52)	0.0056
SF‐6D Score	0.7281	0.1198	0.7236	0.1309	0.7681	0.1284	0.7317	0.1245	0.15 (−0.08;0.37)	0.1897
Total Costs (€)^c^	699.76	1200.83	722.64	1859.60	1715.56	626.30	837.48	1270.68	0.54 (0.38;0.70)	< 0.0001

Abbreviations: BMI, body mass index; CBT, cognitive behavioral therapy group; CI, confidence interval; SD, standard deviation; SF‐6D, Short‐Form 6 Dimensions; WL, waitlist group.

^a^ Pearson's r with 95%‐Confidence interval.

^b^ Mean of the sum of objective and subjective binge‐eating episodes over the past 28 days prior to the assessment time point.

^c^ Standardized costs of the last 4 months prior to baseline or post‐assessment, including the costs of the intervention.

**FIGURE 1:**
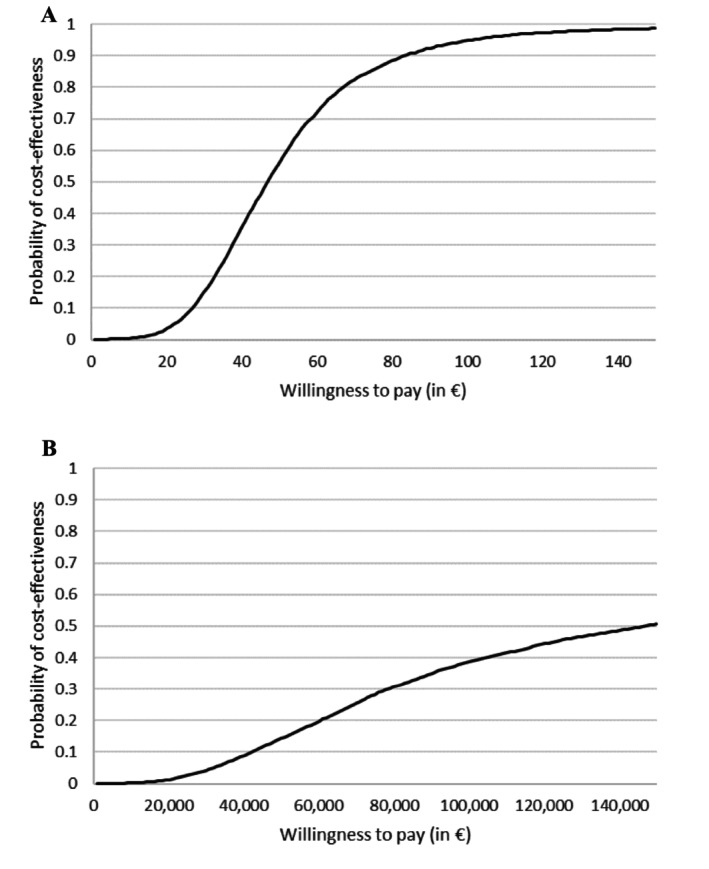
Cost‐effectiveness acceptability curves for the gain of (A) a binge‐free episode or (B) a QALY.

Figure 1A illustrates the probability of cost‐effectiveness for CBT compared to WL, based on the willingness‐to‐pay for the gain of one binge‐free episode. As the willingness‐to‐pay per binge‐free episode increases, the probability of cost‐effectiveness increases in CBT. For a maximum willingness‐to‐pay of €84 for gaining a binge‐free episode, the probability of cost‐effectiveness was 90.3%, rising to 95.0% for a willingness‐to‐pay of €101. Figure 1B illustrates the CEAC for the gain of a QALY. A willingness‐to‐pay of €100,000 for achieving a QALY yielded a probability of cost‐effectiveness of 38.7%. This probability increased to 50.6% for a willingness‐to‐pay of €150,000. CEACs for a billing rate that takes into account costs from 2015, as well as a separate perspective on objective and subjective binge eating, can be found in the Supporting Information.

## 4 Discussion

To the best of our knowledge, this study represents the first cost‐effectiveness analysis of CBT for adolescents with BED. The number of binge‐free episodes was significantly higher in the CBT than the WL group at post‐assessment. As anticipated, the costs associated with CBT were significantly higher, as only the intervention period, during which all intervention costs were incurred, was considered in our analysis. The cost difference is attributed to high intervention costs, while utilization costs were lower in CBT, resulting in an ICER of €46.70 for achieving a binge‐free episode and €128,861 for achieving a QALY. There are currently no established limits defining the maximum willingness‐to‐pay for a binge‐free episode. With regard to costs per QALY, there is no fixed threshold in Germany where a health intervention is considered cost‐effective, as is the case in Great Britain. Overall, the study's findings are likely to empower decision‐makers in the healthcare sector to make evidence‐based determinations regarding the integration of CBT for adolescents with BED into standard clinical practice.

Few studies have examined the cost‐effectiveness of treatments for BED. In a recent study, Melisse et al. (2023) found that web‐based guided self‐help CBT‐enhanced (CBT‐E) was likely cost‐effective compared to a WL control group for adults, with more binge‐free episodes and QALYs gained. König et al. (2018) reported that CBT, compared to an Internet‐based guided self‐help treatment for adults during a 22‐month follow‐up period, was more effective in terms of binge‐free days gained, but also more costly. However, in terms of QALYs gained, CBT was dominated by GSH‐I. Consequently, they did not find clear evidence that one treatment was inherently more cost‐effective.

Among our study's limitations, the sample size of 73 adolescents in this confirmatory RCT is relatively small for a cost‐effectiveness analysis, limiting the ability to detect a significant treatment effect on the HRQoL (Friedman et al. 2015). Multiple imputation was used to ensure that all patients were included. However, missing values increase the uncertainty of the results. As no SF‐6D value set exists for measuring preferences of the German population in healthcare, QALYs were referenced to a representative sample of the UK population, which might differ in preferences (Brazier and Roberts 2004). In a real‐world setting, the intervention would be conducted by psychologists who, unlike those in the study conditions, have no specific training in the BEDA manual. The generalizability of the results may be influenced by the overrepresentation of women, which is a prevalent bias in studies evaluating BED (Hilbert et al. 2019). Finally, this study was conducted a decade ago. It is conceivable that the results would differ today.

## 5 Conclusion

The probability of cost‐effectiveness for the gain of a binge‐free episode was 95.0% at a willingness‐to‐pay threshold of €101, while the probability of cost‐effectiveness for gaining a QALY was 50.6% for a willingness‐to‐pay of €150,000. Unfortunately, due to the WL design, there is no extended follow‐up period to analyze binge‐free episodes, HRQoL, and cost‐effectiveness. The BEDA study (Hilbert et al. 2020) demonstrated stable treatment effects over a 24‐month period, suggesting long‐term reduced costs and improved cost‐effectiveness per QALY, as intervention costs become less significant over time. However, future studies with a longer follow‐up period and a comparison with treatment‐as‐usual or family‐based therapy are needed in order to evaluate the long‐term cost‐utility of CBT in adolescents with BED.

## Author Contributions

**Nicolas Pardey:** formal analysis, methodology, software, validation, visualization, writing – original draft. **Ricarda Schmidt:** data curation, investigation, writing – review and editing. **Jan Zeidler:** methodology, project administration, supervision, writing – review and editing. **Anja Hilbert:** conceptualization, funding acquisition, investigation, project administration, resources, supervision, writing – review and editing.

## Ethics Statement

All procedures performed in studies involving human participants were in accordance with the ethical standards of the institutional and/or national research committee and with the 1964 Helsinki Declaration and its later amendments or comparable ethical standards. Ethical approval for this study was obtained from the Ethical Committee at the University of Leipzig Medical Center.

## Consent

Informed consent was obtained from all participants and at least one parent (if participant < 18 years) for being included in the study. The study was registered at 22th May 2012 at the German clinical trials register (Deutsches Register Klinischer Studien, DRKS, nr: DRKS00000542).

## Conflicts of Interest

Dr. Hilbert reports receiving research grants from the German Federal Ministry of Education and Research, German Research Foundation, Innovation Fund, and Roland Ernst Foundation for Health Care; royalties for books on the treatment of eating disorders and obesity with Hogrefe and Kohlhammer; honoraria for workshops and lectures on eating disorders and obesity and their treatment, including from Lilly; honoraria as editor of the *International Journal of Eating Disorders* and the journal *Psychotherapeut*; honoraria as a reviewer from Oxford University Press and the German Society for Nutrition; and honoraria as a consultant for Takeda. No other conflicts of interest were reported.

## Supporting information


**Data S1.** Supporting Information.

## Data Availability

The data are available upon reasonable request to the senior author.
