# Supplementary material for: Health Economic Evaluation of Cognitive‐Behavioral Therapy for Adolescents With Binge‐Eating Disorder in Germany
Source: Int J Eat Disord. 2025 Mar 10;58(6):1178–83. doi: 10.1002/eat.24413 (PMC12138835; doi:10.1002/eat.24413)

**Supplementary material**

Figure 1 (Supplement) shows the cost-effectiveness acceptability curves for the gain of A) a binge-free episode or B)

a QALY. The costs represent intervention reimbursement for the year 2015, which was 54.70€ at the time of the

study.

**Figure 1 (Supplement): Cost-effectiveness acceptability curves for the gain of A) a binge-free episode or B) a QALY**

**A**

**B**


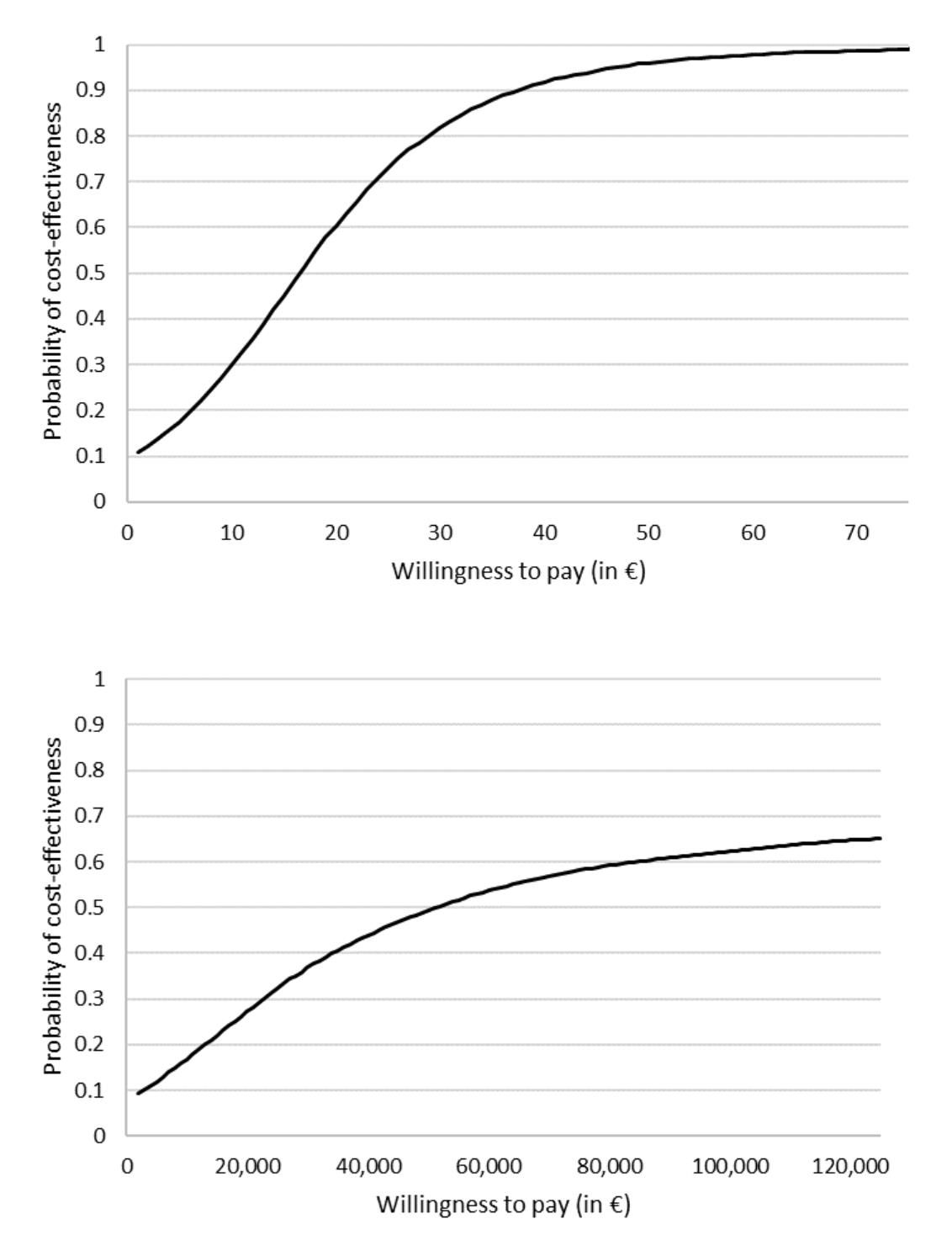


Figure 2 (Supplement) shows the cost-effectiveness acceptability curves for the gain of A) an objective binge-free

episode or B) a subjective binge-free episode. The difference in effectiveness is greater with regard to objective binge-

eating than with subjective binge-eating.

**Figure 2 (Supplement): Cost-effectiveness acceptability curves for the gain of A) an objective binge-free episode or**

**B) a subjective binge-free episode**

**A**

1

0.9

0.8

0.7

0.6

0.5

0.4

0.3

0.2

0.1

0

0

50

100

150

200

Willingness to pay (in €)

**B**

1

0.9

0.8

0.7

0.6

0.5

0.4

0.3

0.2

0.1

0

0

50

100

150

200

Willingness to pay (in €)


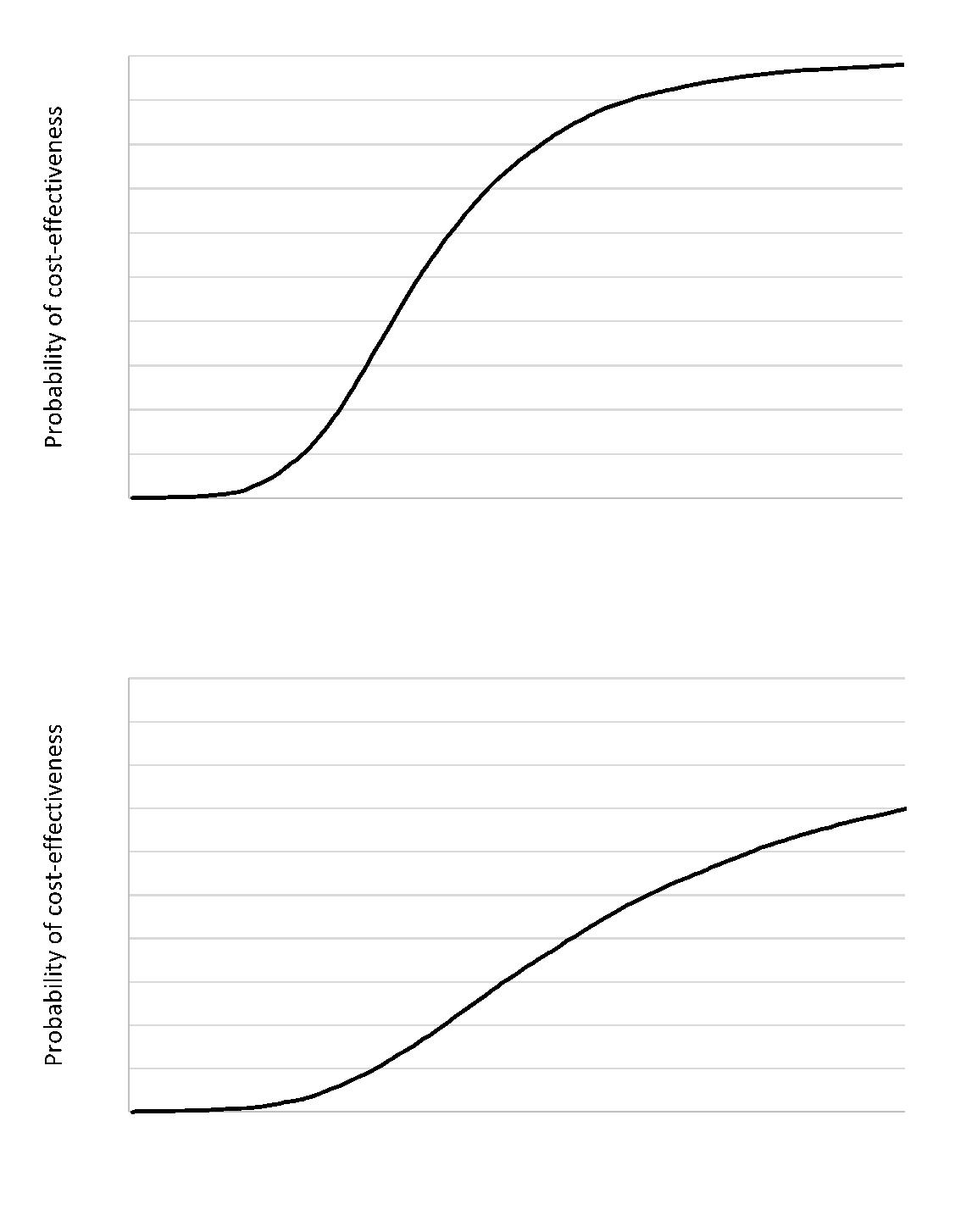

Supplement: Supplementary file 1 — Data S1. Supporting Information. [file EAT-58-1178-s001.docx]
